# Recombinant human endostatin enhances the radioresponse in esophageal squamous cell carcinoma by normalizing tumor vasculature and reducing hypoxia

**DOI:** 10.1038/srep14503

**Published:** 2015-09-28

**Authors:** Hongcheng Zhu, Xi Yang, Yuqiong Ding, Jia Liu, Jing Lu, Liangliang Zhan, Qin Qin, Hao Zhang, Xiaochen Chen, Yuehua Yang, Yan Yang, Zheming Liu, Meiling Yang, Xifa Zhou, Hongyan Cheng, Xinchen Sun

**Affiliations:** 1 Department of Radiation Oncology, The First Affiliated Hospital of Nanjing Medical University, Nanjing 210029, China; 2 Department of Radiation Oncology, Changzhou Cancer Hospital of Soochow University, Changzhou 213001, China; 3 Department of General Internal Medicine, The First Affiliated Hospital of Nanjing Medical University, Nanjing 210029, China

## Abstract

The aim of this study was to investigate the effect of recombinant human endostatin (rh-Endo) in combination with radiation therapy (RT) on esophageal squamous cell carcinoma (ESCC) and explore the potential mechanisms. ECA109-bearing nude mice were administered RT and/or rh-Endo treatment. Tumor volume, survival, hypoxia and vascular parameters were recorded during the treatment schedule and follow-up as measures of treatment response. ESCC cell lines (ECA109 and TE13) and human umbilical vein endothelial cells (HUVECs) were developed to investigate the outcomes and toxicities of rh-Endo and RT *in vitro*. Hypoxia inducible factor-1α (HIF-1α) and vascular endothelial growth factor (VEGF) were also evaluated. *In vivo* studies of ECA109-bearing xenografts showed that rh-Endo improved the radioresponse, with normalization of tumor vasculature and a reduction in hypoxia. *In vitro* studies showed that rh-Endo did not radiosensitize ESCC cell lines but did affect endothelial cells with a time- and dose-dependent manner. Studies of the molecular mechanism indicated that the improved radioresponse might be due to crosstalk between cancer cells and endothelial cells involving HIF and VEGF expression. Our data suggest that rh-Endo may be a potential anti-angiogenic agent in ESCC especially when combined with RT. The improved radioresponse arises from normalization of tumor vasculature and a reduction in hypoxia.

Esophageal cancer (EC) is the eighth most common cancer and the sixth most common cause of cancer-related death in the world, and esophageal squamous cell carcinoma (ESCC) constitutes the major histopathologic subtype^1^. Although radiotherapy (RT) plays an important role in the nonsurgical management of ESCC, radioresistance accounts for a high recurrence rate and poor 5-year survival^2^. In order to reduce the mortality, the development of novel therapeutic strategies is essential.

Angiogenesis is an essential event to allow small, established tumors to grow beyond a critical size of a few millimeters. It is thought that without the necessary microenvironment for neovascularization, tumor growth is arrested^3^. Endostatin is the 20 kDa C-terminal fragment of collagen XVIII, a proteoglycan mainly found in the basement membrane around blood vessels. Endostatin was first described in 1997 by Judah Folkman’s group, and this rapidly drove media attention and Wall Street enthusiasm to EntreMed, the company that licensed its rights^4^. Preclinical and clinical studies reported that recombinant endostatin expressed in bacteria was able to cause the regression of several types of tumor without the induction of resistance and with virtually no signs of toxicity. However, EntreMed abandoned phase III clinical trials because it was unable to provide sufficient quantities of endostatin. Despite halted progress in the western world, endostatin development was pursued in China and recombinant human endostatin (rh-Endo, under the trade name of Endostar) has been approved by the China Food and Drug Administration since 2005. Furthermore, the National Comprehensive Cancer Network (NCCN) Guidelines (Chinese version) have suggested the use of rh-Endo in combination with chemotherapy in patients with non-small cell lung cancer (NSCLC). In addition, several studies have reported that rh-Endo is effective against metastatic melanoma and head and neck cancer^5^,^6^,^7^.

Although previous studies have indicated that rh-Endo may have benefits, it is yet to be established whether rh-Endo might be useful in the treatment of ESCC, particularly when used in combination with RT. The present study utilized both *in vivo* and *in vitro* experiments to investigate the effects of rh-Endo in combination with RT on ESCC, and explore potential mechanisms.

## Results

### rh-Endo enhances the radioresponse in ESCC xenografts

**Figure 1:**
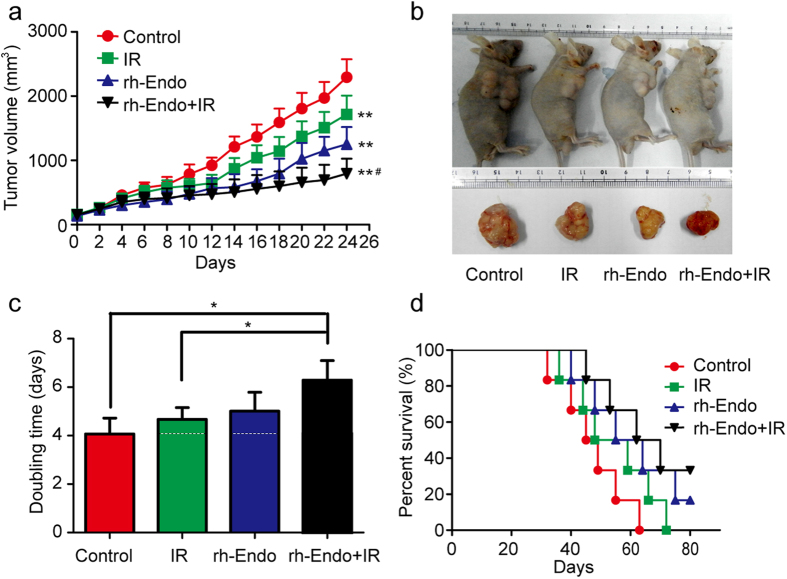
rh-Endo improves the radioresponsiveness of ESCC *in vivo*. ECA109 xenograft-bearing male BALB/c mice were divided into four treatment groups (n = 6): control; IR alone; rh-Endo alone; and a combination of rh-Endo and irradiation. 10 days after inoculation of ECA109 cells (1 × 10^6^ cells/mouse), the mice were irradiated with a single fraction of 8-Gy X-rays. (**a**) Measurements of tumor size. Data represent the mean tumor volume; error bars represent the SD. **P < 0.01 vs. the control group; ^#^*P* < 0.05 vs. the rh-Endo group. (**b**) Representative images of ECA109 xenograft-bearing mice. (**c**) Comparison of tumor doubling times between the four groups. *P < 0.05. (**d**) Kaplan-Meier survival curves for the mice in the four groups.

In the mouse model of ESCC, combined treatment with rh-Endo and irradiation (IR) was more effective at delaying tumor growth than rh-Endo or IR alone (Fig. 1a) (*P* < 0.05). Images of representative cases are shown in Fig. 1b. We further analyzed the time required for the tumor to double in size in each of the treatment groups. The doubling times for the ECA109 tumor in the rh-Endo alone group (5.01 ± 0.66 days) and IR alone group (4.67 ± 1.65 days) were not significantly different from that in the control group (4.07 ± 0.56 days). In contrast, combined treatment with rh-Endo and IR extended the doubling time to 6.79 ± 0.81 days (*P* < 0.05 compared with the control and IR alone groups; Fig. 1c). Intraperitoneal injection of rh-Endo enhanced the response of ECA109 xenografts to IR with an enhancement factor of 2.93 (see Table 1 for details). In addition, there was a trend toward improved survival in mice administered both rh-Endo and IR, although statistical significance was not attained (Fig. 1d).

### rh-Endo normalizes ESCC vascularization

**Figure 2:**
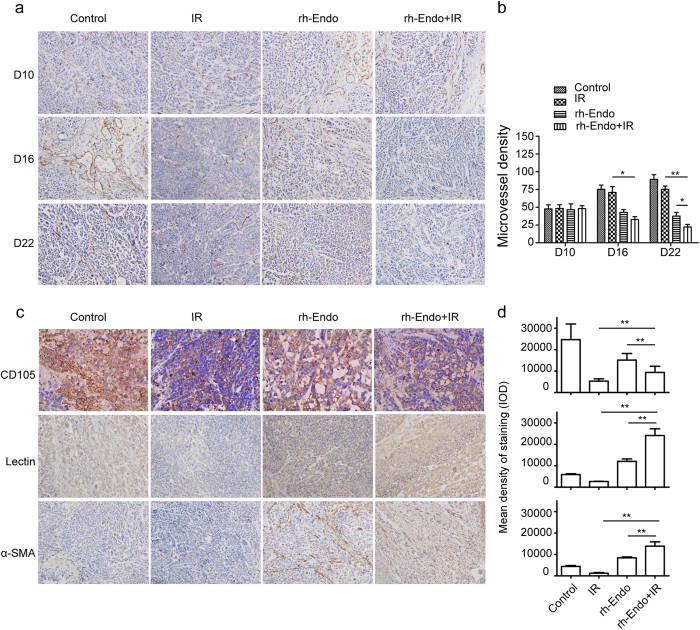
rh-Endo normalizes ESCC vascularization *in vivo*. Tumor tissues from mice in the four treatment groups were removed and stained with anti-CD31, anti-CD105, anti-Lectin and anti-α-SMA antibodies on days 10, 16 and 22 (D10, D16 and D22) after transplantation of ECA109 cells. (**a**) Representative images of CD31 immunostaining (used as a microvessel marker). (**b**) Microvessel densities determined from CD31 immunostaining in the various experimental groups. (**c**) Representative images showing immunostaining for CD105 (a marker of new vessels), Lectin (a marker of functional vessels) and α-SMA (a marker of pericyte coverage). (**d**) Integral optical densities (IODs) of the various experimental groups (upper, CD105; middle, Lectin; lower, α-SMA) on D22 (the end of the treatment period). *P < 0.05 vs. IR group, **P < 0.01 vs. IR group.

Immunohistochemistry (IHC) staining with an antibody directed against CD31 and calculation of micro-vessel density (MVD) were used to detect changes in tumor vascularization and organization on day 10 (D10), day 16 (D16) and day 22 (D22) after transplantation of ECA109 cells. In addition, functional vessels, new vessels and pericyte coverage were assessed using IHC of leptin, CD105 and α-SMA, and the effects of rh-Endo and/or IR treatment were determined on D22. Measurements of CD31 immunoreactivity showed a notable reduction in the vascularity of the ECA109 xenografts after treatment with rh-Endo for 6 days (D16) or 12 days (D22), and an even greater effect of combined therapy (Fig. 2a,b). In further analysis, it was observed that treatment with rh-Endo caused a greater reduction of new vessels than functional vessels, as well as an increase in pericyte coverage (Fig. 2c,d).

### rh-Endo improves ESCC tissue hypoxia

**Figure 3:**
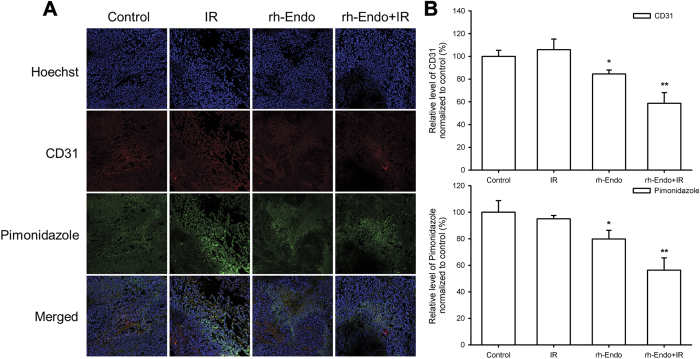
rh-Endo improves ESCC tissue hypoxia *in vivo*. Immunofluorescence assessment of tumor tissues were conducted on D22 (the end of the treatment period). (**A**) Pimonidazole staining showed an improvement of hypoxia in the rh-Endo + IR group. (**B**) Quantify analysis of CD31 and pimonidazole. *P < 0.05 vs. control, **P < 0.01 vs. control.

Interestingly, treatment with rh-Endo significantly improved tumor hypoxia on D22 (i.e. 12 days after treatment), as shown by immunofluorescence (IF) experiments in the ESCC mouse model (Fig. 3A). These data indicate the tumor hypoxia modulation effect of rh-Endo in cells was similar with that in animal models.

### rh-Endo does not improve radiosensitivity *in vitro*

**Figure 4:**
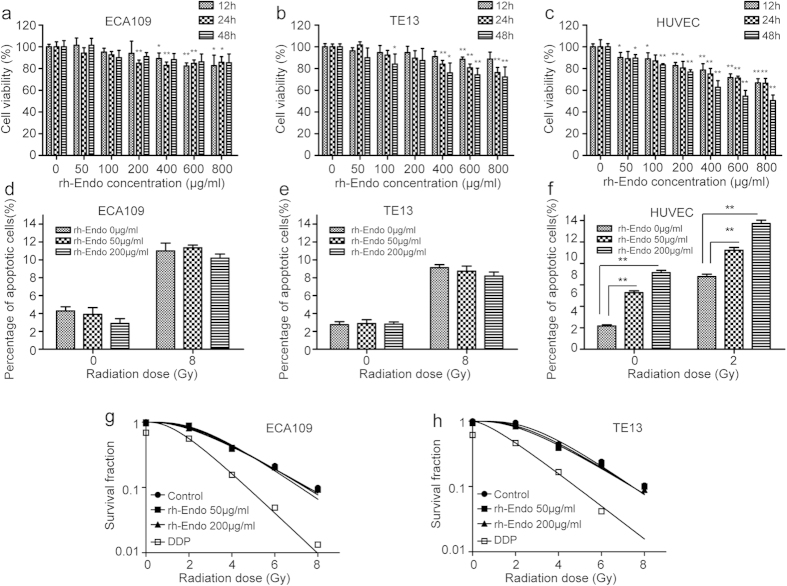
rh-Endo does not improve radiosensitivity of EC cell lines *in vitro* but does affect endothelial cells. (**a–c**) rh-Endo did not inhibit the growth of ECA109 and TE13 cells at lower concentrations (200 μg/mL and below) but did inhibit the growth of HUVECs at these lower concentrations. *P < 0.05 vs. 0 μg/mL at the same time point; **P < 0.01 vs. 0 μg/mL at the same time point. (**d–f**) Flow cytometric analysis revealed that rh-Endo did not induce apoptosis of ECA109 cells and TE13 cells, but did affect the apoptosis of HUVECs. **P < 0.01. (**g,h**) Clonogenic assays showed that rh-Endo did not affect the radioresponsiveness of ESCC cells *in vitro*, whereas DDP improved the sensitivity to radiation.

To determine whether rh-Endo affected ESCC cells directly and whether the improved radioresponse was due to radiation-induced tumor cell death, a cell counting kit (CCK8) assay and colony formation analysis were used. Although higher concentrations of rh-Endo (400 μg/mL and higher) exerted small inhibitory effects on the growth of ESCC cell lines, overall there were no clear time- and dose-dependent effects, as is usually the case for radiosensitizers (Fig. 4a,b). However, rh-Endo did inhibit the growth of human umbilical vein endothelial cells (HUVECs), even at the lowest concentration (50 μg/mL) used (Fig. 4c, *P* < 0.05). Flow cytometry experiments revealed that rh-Endo (at concentrations that did not affect growth of ESCC cell lines) induced apoptosis of HUVECs *in vitro* in a dose-dependent manner (Fig. 4f), but did not induce apoptosis of the ESCC cell lines ECA109 and TE13 (Fig. 4d,e). In colony formation assays, rh-Endo (at concentrations that did not affect growth of ESCC cell lines) also had no effect on ECA109 and TE13 cells, with DDP used as a positive control (Fig. 4g,h). These data suggest that the target of rh-Endo is endothelial cells rather than tumor cells, and that the alleviation of hypoxia was not due to tumor cell killing.

### The improved radioresponse with rh-Endo is associated with decreased expression of HIF-1α and VEGF

**Figure 5:**
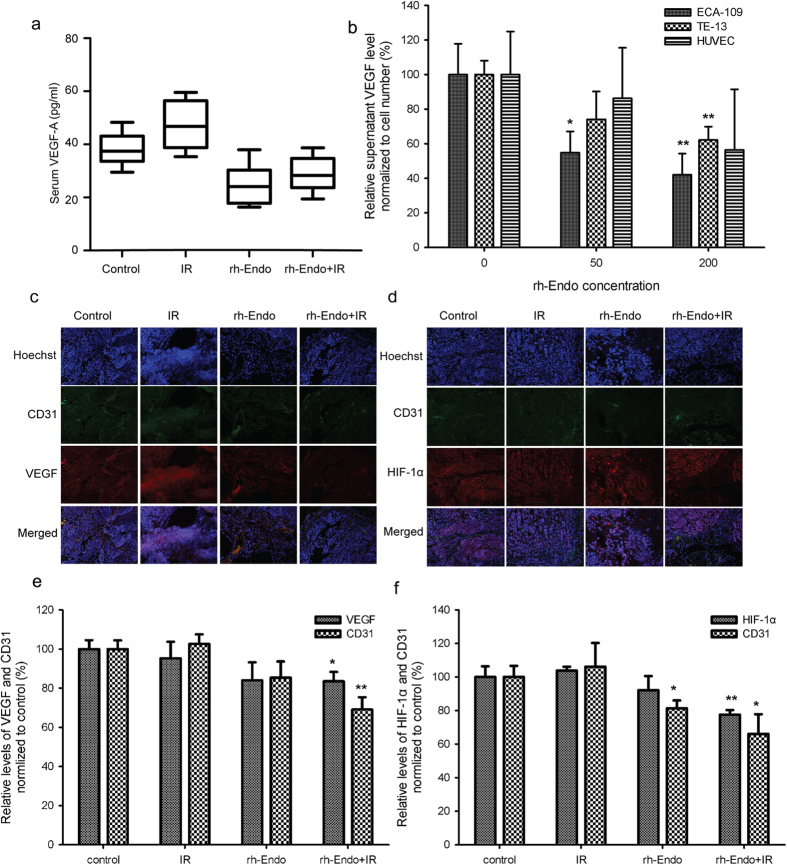
The improved radioresponse with rh-Endo is associated with decreased expression of HIF-1α and VEGF. (**a**) Serum VEGF-A levels measured *in vivo* in the four experimental groups. (**b**) Supernatant levels of VEGF-A from ECA109 cells, TE13 cells and HUVECs normalized to cell numbers. (**c,d**) Immunofluorescence assessment of VEGF and HIF-1α in the four experimental groups on D22. (**e,f**) Quantity analysis of c and d. *P < 0.05 vs. control or 0 μg/mL, **P < 0.01 vs. control or 0 μg/mL.

VEGF expression was evaluated both *in vivo* and *in vitro*. In the animal models, serum VEGF-A was not affected by any of the treatments (Fig. 5a). Meanwhile, the supernatant VEGF-A differed in the medium of ESCC cell lines but not in HUVECs normalized to cell numbers (Fig. 5b). To future evaluate VEGF and HIF-1α expressions in tumor tissues, experiments were performed to detect CD31/VEGF and CD31/HIF-1α. Both VEGF and HIF-1α expressions in the vascular area were reduced when rh-Endo was combined with IR (Fig. 5c–f).

## Disscussion

RT is a well-established therapeutic modality in oncology. It provides survival benefits in several cancer types, including EC^8^. rh-Endo is approved by the China Food and Drug Administration and its use as an anti-angiogenic agent in advanced NSCLC is suggested in the NCCN guidelines (Chinese version)^9^. Moreover, clinical trials and retrospective studies have demonstrated an anti-tumor effect of rh-Endo in metastatic melanoma, head and neck cancer, breast cancer and gastric cancer^10^. However, there remains debate as to whether rh-Endo can be used in EC.

This is the first comprehensive report of the use of rh-Endo with RT in EC. To explore the intrinsic mechanism underlying the contribution of angiogenesis inhibition to the radioresponse in EC, we used *in vivo* studies of xenografts and *in vitro* cell line models. Translational experiments indicated that the improved radioresponse with rh-Endo might be attributable to tumor vasculature remodeling and hypoxia reduction, possibly through regulation of the HIF/VEGF pathway. rh-Endo does not kill EC cells directly, but likely affects crosstalk between endothelial cells and cancer cells, such as VEGF-A expression, which directly affects endothelial cell proliferation^11^.

The intrinsic mechanisms underlying the contribution of angiogenesis inhibitors to the radioresponse remain unclear, despite a considerable amount of research in recent years. Some authors have suggested that angiogenesis inhibitors and RT synergistically enhance the radioresponse of tumor growth in solid tumors^11^,^12^,^13^. Importantly, there is evidence in different tumors that angiogenesis inhibitors can induce vascular normalization^14^,^15^. Notwithstanding these findings, new insights are needed to explain the potential mechanism that may be operative in the improved radiation response with angiogenesis inhibitors.

Aberrant tumor vasculature is hyperpermeable and tortuous, and confers compromised blood supply to tumor tissues leading to a hypoxic and acidic tumor microenvironment^16^,^17^. We observed vascular normalization in ESCC xenografts 5 days after therapy with rh-Endo. As might be expected, we observed the most significant delay in tumor growth when IR was given on day 5, with an effect superior to that of rh-Endo alone, IR alone, and IR performed 1 day before or after rh-Endo (data not shown) in mice. In further studies, rh-Endo was found to affect both CD105 and lectin, markers of new vessels and functional vessels. In contrast to the reduced MVD, we observed an increase in the amount of pericytes and their endothelial coverage 5 days after rh-Endo treatment (IHC of α-SMA). An increased number of pericytes and restored close pericyte-endothelial cell interactions have been observed almost universally in studies showing vascular normalization^18^.

Hypoxic tumors have long been demonstrated to possess higher radiation resistance, and numerous modalities have attempted to address this by improving the oxygen supply^18^. In our study, pimonidazole was employed to validate the hypoxic change, and rh-Endo was found to reduce overall tumor hypoxia from day 5. Excitingly, the modulation of hypoxia on day 5 after rh-Endo treatment provides a basis for the design of an rh-Endo/IR combination schedule in future clinical practice.

It is currently not clear how anti-angiogenic treatment normalizes the aberrant tumor vasculature and the underlying mechanism involving hypoxic changes. *In vitro* studies were conducted to determine whether rh-Endo radiosensitizes ESCC cells directly, and these showed no radiosensitization effect in ESCC cell lines. However, rh-Endo was able to influence the proliferation and apoptosis of endothelial cells directly. In the molecular analysis, we detected HIF/VEGF expression changes in the tissues after treatment with rh-Endo, and this was enhanced by irradiation. Treatment did not affect serum VEGF-A expression in xenografts and HUVECs, but did affect VEGF-A secretion by ESCC cell lines. VEGF-A is well established as a regulator of the proliferation, migration and survival of endothelial cells^19^. This suggests that VEGF secreted by cancer cells may have an impact on endothelial cells, which can further contribute to change in tumor vasculature and hypoxia^20^.

There are some limitations to this study. First, the ECA109 cell line was chosen because it is highly proliferative, representative, and is resistant to many therapies. However, the beneficial effects of rh-Endo on IR need to be confirmed in other cell lines. Second, a single large-dose of IR was employed to exclude the potential confounding effects of conventional fractionated IR. However, the use of a single 8-Gy dose, selected on the basis of previous studies^21^,^22^, differs from regimens that are used clinically. Therefore, the optimal combination of rh-Endo with conventional IR warrants further investigation. Third, measurements of tumor volume were made only up to D22; although this observation period was based on that used in previous investigations^23^,^24^, additional research is warranted to determine the effects of rh-Endo and IR on tumor volume over extended periods. Fourth, additional studies in eligible patients with ESCC are needed to explore changes in tumor hypoxia and vasculature after treatment with rh-Endo and RT. Fifth, although there appeared to be a trend for improved survival in mice treated with both rh-Endo and IR, statistical significance was not attained. It may be that our study was underpowered (n = 6 per group) to detect any real differences in survival between groups; thus additional studies with larger sample sizes are merited.

These findings indicate that rh-Endo is a potential anti-angiogenic agent in ESCC especially when combined with RT. The improved radioresponse may arise from normalization of the tumor vasculature and a reduction in tumor hypoxia^25^,^26^. Our data support the emerging notion that the HIF/VEGF pathway may be involved in the improved radioresponse. However, clinical studies are needed to further confirm these findings.

## Materials and Methods

### Cell lines, cell culture and reagents

ESCC cell lines ECA109 and TE13 were obtained from Shanghai Institute of Cell Biology (Shanghai, China) and were maintained in DMEM medium (Gibco, Life Technologies, Carlsbad, CA, USA) supplemented with 10% fetal bovine serum (Hyclone, GE Healthcare, Little Chalfont, UK), 1% penicillin/streptomycin (Invitrogen, Life Technologies). Cells were maintained in an incubator at 37 °C, in an atmosphere containing 5% CO_2_. HUVECs were isolated from human umbilical cord veins using a standard procedure described previously^27^, and grown in EBM-2 medium with SingleQuots™ (Lonza, Walkersville, MD, USA) containing VEGF and other growth factors.

Cells in the IR group were subjected to a 2, 4, 6 or 8 Gy of X-ray irradiation from a medical linear accelerator (Elekta Precise) at room temperature.

anti-CD31, anti-CD105, anti-CD31, anti-CD105, anti-lectin, anti-α-SMA, anti-VEGF, anti-HIF-1α (Abcam), fluorescein (FITC)-conjugated anti-mouse IgG, and horseradish peroxidase (HRP)-conjugated anti-rabbit IgG (Jackson ImmunoResearch, West Grove, PA, USA). rh-Endo was provided by Simcere Pharmaceuticals (Nanjing, China).

### Xenografts

All animal experiments were approved by the Ethics Committee of Nanjing Medical University, and performed in accordance with the guidelines of the National Institutes of Health and Nanjing Medical University. Male BALB/C nude mice (age: 8 weeks; weight: 19.04 ± 1.59 g) were provided by Nanjing Medical University Animal Center, and xenografts were established in each mouse by subcutaneous injection of 0.1 mL of ECA109 cells (1 × 10^7^ cells/mL) into the right proximal hind leg.

After 9 days of incubation, when the tumors had reached approximately 100–150 mm^3^ in volume, the animals were assigned into 1 of 4 groups (n = 6 per group): (1) vehicle (saline, D10–D22), (2) rh-Endo (D10–D22), (3) IR (D16), (4) rh-Endo (D10–D22) +IR (D16). rh-Endo was administered by injection via the caudal vein at a dose of 15 mg/kg, once daily for 13 days. The control (vehicle) group was administered similar volumes of physiologic saline. Tumors were irradiated with X-rays (8 Gy dose, 2 Gy/min) on D16, using an RS-2000 biological irradiator. The selection of a single dose of 8-Gy of X-rays was made on the basis of previous studies using single doses of 2–20 Gy^21^,^22^. Tumor growth was measured every day until D25, and the tumor volume was calculated according to the formula: Tumor volume = (length [L] × width [W])^2^/2. Mice were raised until they died naturally, and survival time was recorded.

Based on the previous results, another 72 xenografts were allocated into the same 4 groups (n = 18 per group) as described above. Six mice in each group were killed on day 10 (before rh-Endo), day 16 (before IR) and day 22 (after the treatment schedule). Pimonidazole (Hypoxyprobe kit, #HP1-100; Chemicon International, Schwalbach, Germany), a hypoxia marker, and Hoechst 33342 (B2261; Sigma-Aldrich, St. Louis, MO, USA), a perfusion marker, were injected 60 min before the mouse was killed.

### Immunohistochemistry (IHC)

Paraffin-embedded tumor tissue sections were deparaffinized in xylene, rehydrated in graded ethanol, and rinsed twice with phosphate-buffered saline (PBS). Endogenous peroxidase activity was blocked by incubating sections with 3% hydrogen peroxide in the dark for 15 min. The sections were then incubated overnight at 4 °C with polyclonal antibody to CD31, CD105, lectin or α-SMA (diluted 1:500). After washing, slides were incubated with HRP-conjugated anti-rabbit secondary antibody (diluted 1:100) for 1 h at room temperature. Finally, the slides were visualized by incubation with 3, 3′-diaminobenzidine (DAB) (Dako, Hamburg, Germany) and counterstained with hematoxylin (37%). The sections were analyzed under an Axiovert A1 light microscope (Zeiss, Jena, Germany). The intensity of the immunoreactivity in the tumor was evaluated using Image-Pro Plus 6.0 software. Mean intensity was calculated by dividing the integral optical density (IOD) by the area^28^. The microvessel density was calculated as a ratio of microvessel number and area of the picture with Image pro-plus 6.0 software.

### Immunofluorescence (IF)

The tissues were fixed with acetone and permeabilized with 0.1% Triton X-100 in PBS for 5 min at room temperature. The tissues were then incubated with antibodies against Hoechst, CD31, pimonidazole, HIF-1α, and VEGF (diluted 1:500) at 4 °C overnight, and then with FITC-conjugated secondary antibody (diluted 1:100) for 1 h at room temperature. After washing in PBS, cells were incubated with 0.25 mg/mL DAPI for 3 min. The images were examined using a laser scanning confocal microscope (LSM510, Zeiss).

### Cell viability assay

Cell proliferation was measured using a CCK8 assay. Cells were plated at a concentration of 5 × 10^3^ cells/well in 96-well plates. The medium was removed and replaced with fresh medium with or without rh-Endo after 24 h. A CCK8 cell proliferation and cytotoxicity assay kit (Obio Technology, Shanghai, China) was used after 12, 24 and 48 h. The absorbance was measured at a wavelength of 490 nm.

### Flow cytometric analysis

ECA109, TE13 and HUVEC cells were fixed in 2% paraformaldehyde and then stained with an Annexin V-FITC Apoptosis Kit (Keygene Biotechnology, Nanjing, China). The cells were analyzed by flow cytometry using FACSan with Cell-Quest software (BD Biosciences, San Jose, CA, USA)^29^.

### Clonogenic survival assay

ECA109 and TE13 cells were seeded onto six-well dishes. After overnight culture, the cells were treated with rh-Endo (50 μg/mL or 200 μg/mL) for 24 h. Cells treated with 10 μM cisplatin (DDP) were used as positive controls, and a negative control group was also used. Cells were then irradiated with 6MV X-rays at doses of 0, 2, 4, 6 or 8 Gy at 4.5 Gy/min. The cells were then cultured in a 5% CO_2_ incubator at 37 °C for 10 days. The colonies were fixed and stained with crystal violet to count the number of colonies (>50 cells/colony).

### VEGF-A assay

The amount of VEGF-A in the culture medium and serum of xenografts was measured with a commercial ELISA kit (R&D Systems, Minneapolis, MN, USA).

### Data analysis

The mean ± standard deviation (SD) from triplicate assays was calculated, and differences between treatment groups were determined using the ANOVA test. Statistical analysis was performed using STATA 11.0 software (StataCorp, College Station, TX, USA) and Prism 5.0 software (GraphPad, La Jolla, CA, USA). P < 0.05 was considered statistically significant.

## Additional Information

**How to cite this article**: Zhu, H. *et al.* Recombinant human endostatin enhances the radioresponse in esophageal squamous cell carcinoma by normalizing tumor vasculature and reducing hypoxia. *Sci. Rep.* **5**, 14503; doi: 10.1038/srep14503 (2015).

## Figures and Tables

**Table 1:** The growth of ECA109 xenografts in different groups of mice.

**Treatment**	**Doubling time (days)**	Absolute growth delay (days)*	**Normalized growth delay (days)**†	**Enhancement factor**
Control	4.07 ± 0.56			
rh-Endo	5.01 ± 0.66	0.94 (5.01-4.07)		
IR	4.67 ± 1.65	0.60 (4.67-4.07)		
IR + rh-Endo	6.79 ± 0.81	2.72 (6.79-4.07)	1.76 (2.72-0.94)	2.93 (1.76/0.60)

IR, irradiation.

^*^Tumor doubling time in the treatment groups minus that of the control group.

^†^Subtraction of the absolute growth delay of the tumor in the group treated with rh-Endo alone from that of the group treated with both irradiation and rh-Endo.
